# Abnormal gastric electrophysiology following laparoscopic sleeve gastrectomy and associations with symptoms and quality of life

**DOI:** 10.1093/bjsopen/zraf140

**Published:** 2025-12-01

**Authors:** Tim Hsu-Han Wang, Chris Varghese, Sam Robertson, Grant Beban, Nicholas Evennett, Daphne Foong, Vincent Ho, Christopher N Andrews, Stefan Calder, Armen Gharibans, Gabriel Schamberg, Greg O'Grady

**Affiliations:** Department of Surgery, The University of Auckland, Auckland, New Zealand; Department of Surgery, Auckland City Hospital, Auckland, New Zealand; Department of Surgery, The University of Auckland, Auckland, New Zealand; Department of Surgery, The University of Auckland, Auckland, New Zealand; Department of Surgery, Auckland City Hospital, Auckland, New Zealand; Department of Surgery, Auckland City Hospital, Auckland, New Zealand; School of Medicine, Western Sydney University, Sydney, New South Wales, Australia; School of Medicine, Western Sydney University, Sydney, New South Wales, Australia; Department of Gastroenterology and Hepatology, Campbelltown Hospital, Sydney, New South Wales, Australia; Division of Gastroenterology and Hepatology, University of Calgary, Calgary, Alberta, Canada; Department of Surgery, The University of Auckland, Auckland, New Zealand; Alimetry Ltd, Auckland, New Zealand; Department of Surgery, The University of Auckland, Auckland, New Zealand; Alimetry Ltd, Auckland, New Zealand; Auckland Bioengineering Institute, The University of Auckland, Auckland, New Zealand; Department of Surgery, The University of Auckland, Auckland, New Zealand; Alimetry Ltd, Auckland, New Zealand; Department of Surgery, The University of Auckland, Auckland, New Zealand; Alimetry Ltd, Auckland, New Zealand; Auckland Bioengineering Institute, The University of Auckland, Auckland, New Zealand

**Keywords:** gastric myoelectrical activity, high-resolution electrogastrography, slow waves, Gastric Alimetry, body surface gastric mapping

## Abstract

**Background:**

Sleeve gastrectomy is an effective bariatric procedure but may lead to persistent symptoms without obvious mechanical cause. The normal gastric pacemaker region, which lies on the greater curvature of the corpus, is resected in sleeve gastrectomy, but the electrophysiological consequences are not adequately defined. This study assessed these impacts and associations with symptoms and quality of life (QoL) using non-invasive gastric mapping.

**Methods:**

Patients with previous sleeve gastrectomy underwent body surface gastric mapping (Gastric Alimetry), comprising 30-minute fasting baseline and 4-hour post-prandial recordings. Analysis encompassed principal gastric frequency (PGF), body mass index-adjusted amplitude, and the Gastric Alimetry Rhythm Index (GA-RI), with comparison to reference intervals and matched controls. Symptoms were evaluated using a validated app and questionnaires.

**Results:**

The study recruited 38 patients (median 36 months after surgery; range 6–119 months) and 38 controls. Of the 38 patients, 35 had at least one abnormal parameter compared with controls, typically reduced frequencies (mean(standard deviation) 2.30(0.34) *versus* 3.08(0.21) c.p.m., respectively; *P* < 0.001) and amplitudes (14.8(6.9) *versus* 31.5(18.0) µV, respectively; *P* < 0.001). Patients exhibited higher symptoms and lower QoL than the controls (Patient Assessment of Upper Gastrointestinal Disorders (PAGI) Symptoms Questionnaire scores 20 *versus* 7, respectively (*P* < 0.001); PAGI-QOL 27 *versus* 136, respectively (*P* < 0.001)). Gastric amplitude (*R* = 0.71, *P* < 0.001) and the GA-RI (*R* = 0.60, *P* = 0.02) were positively correlated with bloating, whereas amplitude was negatively correlated with heartburn (*R* = −0.46, *P* = 0.03). Lower gastric amplitudes were also correlated with greater weight loss (*R* = −0.45; *P* = 0.014).

**Conclusion:**

Sleeve gastrectomy modifies gastric electrophysiology due to pacemaker resection, with variable remodelling. Substantial reductions in gastric frequency and amplitude occur routinely after surgery, with specific associations between post-procedural gastric amplitude and symptoms of heartburn, bloating, and weight loss identified.

## Teaser

This paper explores how sleeve gastrectomy, a bariatric procedure, affects gastric electrophysiology. The study used non-invasive gastric mapping to compare electrical activity in patients after surgery with that in a control group. Key findings show the procedure alters gastric electrophysiology, often reducing gastric frequencies and amplitude, and that these changes correlate with symptom development and weight loss.

## Introduction

Sleeve gastrectomy is a common procedure for obesity and its associated consequences, including diabetes and metabolic syndrome. A significant portion of the greater curvature of the stomach is resected from the antrum to the fundus, reducing gastric volume^1^. Although most people experience excellent outcomes in terms of weight loss, metabolic outcomes, and quality of life (QoL)^2^, up to 30% develop persistent foregut symptoms such as nausea, postprandial bloating, heartburn, and reflux^3–7^. These symptoms are commonly evaluated endoscopically and radiologically and are usually managed medically. However, a subset of patients experiences chronic symptoms unrelated to gastro-oesophageal reflux disease despite normal investigations and no evident mechanical cause. These symptoms are distressing for the patients and therefore impact on their QoL.

Gastric motility is controlled by a gastric pacemaker region located at the greater curvature of the upper corpus, from which slow waves propagate circumferentially then antegrade, at a frequency close to 3 cycles per minute (c.p.m.), before rapidly accelerating in the terminal antrum^8–11^. This normal pacemaker region is completely resected during a sleeve gastrectomy procedure, but the implications for gastric motility and post-surgical symptoms remain poorly understood^12,13^.

A new non-invasive technique for monitoring gastric motility has recently emerged, called body surface gastric mapping (BSGM), with a commercial device called Gastric Alimetry (Alimetry, Auckland, New Zealand) recently achieving clinical translation and regulatory clearances^14,15^. This new test also includes an App that allows simultaneous validated symptom profiling, enabling robust symptom correlation with gastric electrical abnormalities^15^. Recent studies using this technology are promising, revealing a range of slow wave initiation and conduction abnormalities in medical and surgical conditions^16–22^.

The aim of the present exploratory study was to assess the long-term effects of sleeve gastrectomy on gastric function measured by BSGM, and their contributions to persistent gastric symptoms and postoperative QoL.

## Methods

Ethics approval for this study was granted by the institutional review committees of The University of Auckland, Western Sydney University, and The University of Calgary Conjoint Health Research Ethics Board (AH1125, H15157, and REB19-1925). Consecutive eligible patients who underwent a sleeve gastrectomy within the past 10 years were then recruited from the surgical database from these institutions. Patients were excluded if they had a history of skin allergy to adhesives or evidence of mechanical gastric or small bowel obstruction as a cause of their symptoms. Informed consent was obtained from all patients. A healthy matched control cohort was selected, originally part of a previous study^23^, who had no significant history of gastric disorders, gastric symptoms, gastrointestinal procedures, diabetes, or medications affecting gastrointestinal function.

### Body surface gastric mapping

**Fig. 1:**
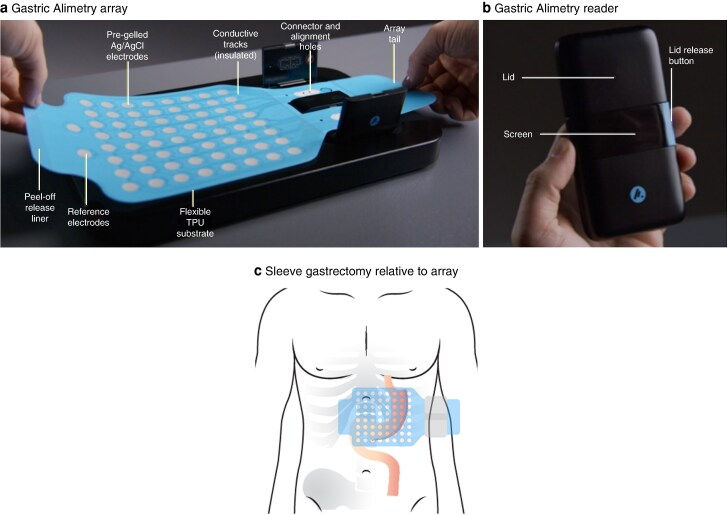
Gastric Alimetry array, Gastric Alimetry reader, and diagram showing sleeve gastrectomy in relation to array placement **a** Gastric Alimetry array, **b** Gastric Alimetry reader, and **c** diagram of the sleeve gastrectomy relative to array placement.

Gastric Alimetry was performed under a protocol adapted for patients with a reduced gastric volume. The Gastric Alimetry device comprises a high-resolution stretchable electrode array (8 × 8 electrodes; 20-mm spacing; 196 cm^2^), a wearable Reader, a validated iOS app for symptom logging, and a cloud-based reporting platform (*Fig. 1a, b*)^24–26^. Baseline recordings were performed in the first 30 minutes followed by a 218-kcal meal comprising 100 ml Ensure^®^ liquid meal (93 kcal; Abbott Nutrition, Abbott Park, IL, USA) and half an oatmeal energy bar (125 kcal; 2.5 g fat, 22.5 g carbohydrate, 5 g protein, and 3.5 g fibre; Clif Bar, Emeryville, CA, USA), consumed over 10 minutes, and a 4-hour postprandial recording in order to capture a full gastric activity cycle. Patients were seated in a reclined chair and were asked to limit movement, talking, and sleeping, but were able to read, watch media, work on a mobile device, and mobilize for comfort breaks^27^. The matched controls underwent the standard Gastric Alimetry test using the standard 482 kcal meal (200 ml Ensure^®^ liquid meal and one whole oat meal energy bar (Clif Bar))^27^. The impact of different test meal sizes between the two groups is known, having been rigorously analysed in a separate study quantifying modest reductions in postprandial amplitudes and rhythm index with smaller meal sizes^28^.

Spectral analysis was performed, encompassing the four established test metrics^29^: principal gastric frequency (PGF; c.p.m.), body mass index (BMI)-adjusted amplitude (µV), Gastric Alimetry Rhythm Index (GA-RI; reflecting pacemaker stability), and the fed:fasted amplitude ratio (ff-AR; indicating meal response with contractions). These metrics were assessed using published normal ranges^23^. Frequency was not reported if a stable gastric rhythm was not detected^25^. Adverse events were assessed and recorded.

### Symptom and QoL assessments

Symptom development during the test was recorded on the validated Gastric Alimetry app and summarized as a total symptom burden score (TSBS)^26^. Symptoms and QoL over the preceding 2 weeks were further assessed using validated questionnaires, namely the Patient Assessment of Upper Gastrointestinal Disorders Symptoms questionnaire (PAGI-SYM), the Patient Assessment of Upper Gastrointestinal Disorders—Quality of Life questionnaire (PAGI-QOL), and the EQ-5D-5L^30–32^.

### Statistical analyses

Data were analysed using GraphPad Prism (GraphPad Software, San Diego, CA, USA) and R v.4.0.1 (R Foundation for Statistical Computing, Vienna, Austria). Spectral analysis comparisons with the age- and sex-matched healthy controls were performed using unpaired Student’s *t* tests. All data are presented as the mean(standard deviation). Pearson’s correlation analyses were performed between the test spectral metrics, symptoms, and QoL scores. A threshold of a two-tailed *P* < 0.050 was deemed to be statistically significant.

## Results

In all, 38 patients were recruited to the study (12 male, 26 female). The median age of the patients was 51.7 years (range 22–73 years), median BMI was 40.9 kg/m^2^ (range 29.5–65.8 kg/m^2^) at the time of operation and 33.0 kg/m^2^ (range 20.4–47.3 kg/m^2^) at the time of study participation. The median duration since surgery was 46.2 months (range 6–119 months). Morbid obesity was the indication for all sleeve gastrectomies. The demographic characteristics of the patient and matched control groups are presented in *Table 1*. Compared with the patient group, the matched controls had a lower BMI (33.0(6.0) *versus* 27.4(8.0) kg/m^2^; *P* < 0.001) and were slightly younger (51.7(11.8) *versus* 44.0(14.6) years; *P* = 0.01). It should be noted that the Gastric Alimetry metrics, including both the amplitude and GA-RI, are adjusted for BMI^29^.

**Table 1:** Demographic, QoL, and spectral characteristics for patients and matched controls

Characteristics	Patients (*n* = 38)	Matched controls (*n* = 38)	P*
Age	51.7(11.8)	44.0(14.6)	0.01
BMI (kg/m^2^)	33.0(6.0)	27.4(8.0)	< 0.001
**Sex (*n*)**			
Male	12	11	1
Female	26	27	
Time since surgery (months)	46.2(32.8)	N/A	
**QoL**			
TSBS	5.9(7.4)	2.7(7.0)	0.06
PAGI-SYM	20(19)	7(14)	< 0.001
PAGI-QOL	27(29)	136(20)	< 0.001
EQ-5D-5L	0.86(0.19)	0.96(0.09)	< 0.01
Self-reported EQ-5D-5L	74(21)	88(8)	< 0.001
**Spectral characteristics**			
Frequency (2.65–3.35 c.p.m.)	2.30(0.34)	3.08(0.21)	< 0.001
Adjusted amplitude (22–70 µV)	14.8(6.9)	31.5(18.0)	< 0.001
ff-AR (≥ 1.08)	1.60(0.54)	2.00(0.92)	0.02
GA-RI (≥ 0.25)	0.35(0.14)	0.50(0.20)	< 0.001

Data are given as the mean(standard deviation), unless otherwise stated. QoL, quality of life; BMI, body mass index; TSBS, total symptom burden score; PAGI-SYM, Patient Assessment of Upper Gastrointestinal Disorders Symptoms questionnaire; PAGI-QOL, Patient Assessment of Upper Gastrointestinal Disorders—Quality of Life questionnaire; ff-AR, fed:fasted amplitude ratio; GA-RI, Gastric Alimetry Rhythm Index. *Student's t test and the χ2 test was used for data analysis.

### High-resolution gastric mapping

**Fig. 2:**
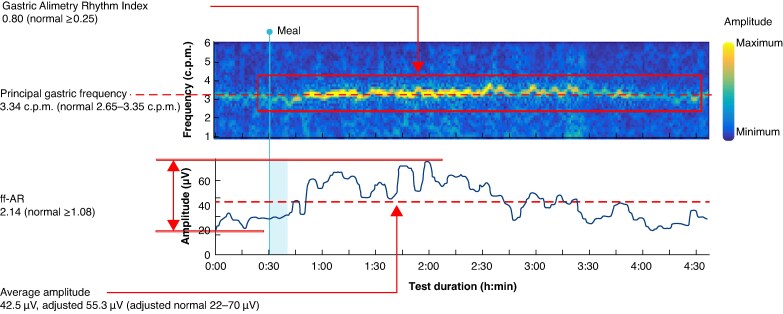
Representative spectral diagram for a healthy matched control Normative values are provided in parentheses. h, hour; min, minute; ff-AR, fed : fasted amplitude ratio; c.p.m., cycles per minute.

**Fig. 3:**
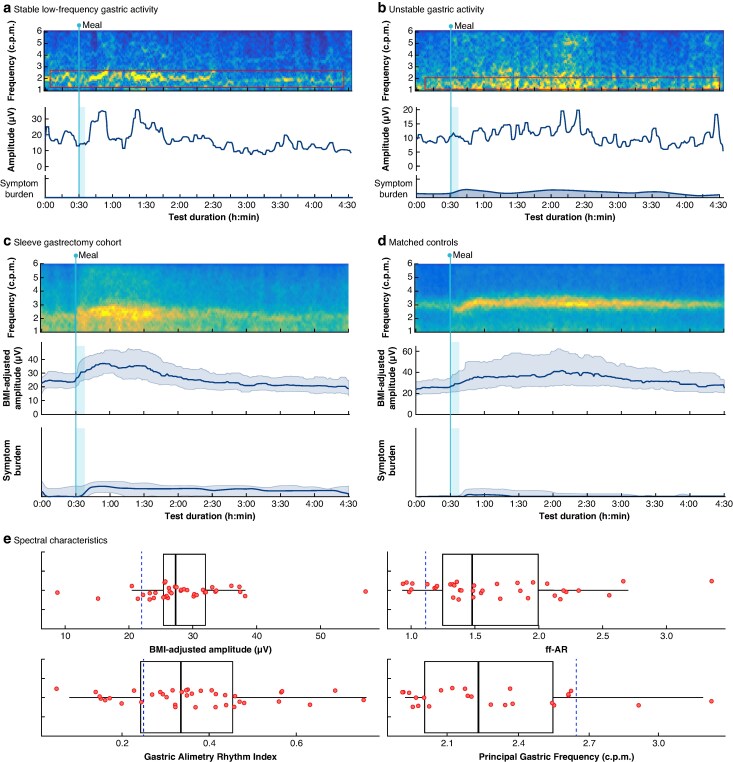
Representative spectral diagrams from patients with abnormal spectral characteristics and symptoms, mean spectrograms for the sleeve gastrectomy and matched cohorts, and summary spectral characteristics for the entire cohort **a** Spectral diagram for a patient with stable low frequency gastric activity. **b** Spectral diagram for a patient with unstable gastric activity with low amplitude and moderate symptom development. The boxed red lines in **a** and **b** indicate the degraded principal gastric frequency; however, these are significantly lower in amplitude than in healthy patients. Mean spectrogram, median BMI-adjusted amplitude, and total symptom burden score for the **c** sleeve gastrectomy cohort and **d** matched control cohort (*n* = 38 in each). Shaded areas represent the interquartile range. **e** Box-and-whisker diagrams of BMI-adjusted amplitude, ff-AR, Gastric Alimetry-Rhythm Index, and Principal Gastric Frequency. The boxes show the interquartile range, with the median value indicated by the vertical line; whiskers show the range. Dashed blue lines denote the lower limit of the normal range. c.p.m., cycles per minute; h, hour; min, minute; BMI, body mass index; ff-AR, fed : fasted amplitude ratio.

Of the 38 patients, 35 had at least one abnormal gastric slow wave activity parameter compared with the normative test reference range (*Table 1*). Of the 24 patients with a detectable PGF, 21 had a reduced frequency (mean 2.30 c.p.m.). The remaining 14 patients had an undetectable frequency due either to very irregular gastric rhythms or an inability to detect remnant gastric activity cutaneously. Of the 38 patients, 12 had two abnormal gastric activity characteristics, including a combination of an abnormal principal gastric frequency with a low GA-RI (3 patients), ff-AR (8 patients), or BMI-adjusted amplitude (4 patients). Overall, four patients were found to have a reduced BMI-adjusted amplitude, eight had a reduced ff-AR, and 10 had a reduced GA-RI. An representative spectral map from a healthy matched control is shown in *Fig. 2*, together with normative reference interval ranges, whereas two typical examples of patient spectral maps are shown in *Fig. 3a, b*.

When the pooled results were compared between the patients and the matched controls (*Table 1*), statistically significant differences were found in all gastric slow wave characteristics: frequency, 2.30 *versus* 3.08 c.p.m., respectively (*P* < 0.001); BMI-adjusted amplitude, 14.8 *versus* 31.5 µV, respectively (*P* < 0.001); ff-AR, 1.60 *versus* 2.00, respectively (*P* = 0.02); and GA-RI, 0.35 *versus* 0.50, respectively (*P* < 0.001). Averaged spectrogram data for visual whole-cohort comparisons are shown in *Fig. 3c, d*. Visual analysis of the averaged spectrograms of patients *versus* controls also demonstrates how gastric pacemaker rhythm became significantly more variable after sleeve gastrectomy, with a wider, shorter, and more irregular gastric spectral activity band (*Fig. 3c, d*).

No adverse events occurred due to body surface gastric mapping testing.

### QoL, symptom, and weight loss evaluations

The QoL data and TSBS are presented in *Table 1*. Overall, 14 of 38 patients had significant symptoms during the test, as judged by a TSBS ≥ 5. Seven of these patients had substantial symptoms, with a TSBS ≥ 10. The mean TSBS in the patient cohort was 5.9, *versus* 2.6 in the matched controls (*P* = 0.06; *Table 1*). Compared with the matched controls, the patient cohort also had higher symptom burdens and a substantially reduced QOL, as assessed using the PAGI-SYM (7 *versus* 20, respectively; *P* < 0.001), PAGI-QoL (136 *versus* 27, respectively; *P* < 0.001), EQ-5D-5L (0.96 *versus* 0.86, respectively; *P* < 0.01), and self-reported EQ-5D-5L (88 *versus* 74, respectively; *P* < 0.001; *Table 1*). Predominant symptoms included postprandial excessive fullness and aversion to food.

Results of correlation analyses between the gastric mapping parameters and individual symptom measures are presented in *Table 2* and *Fig. S1*. The BMI-adjusted amplitude was positively correlated with bloating (*R* = 0.71, *P* < 0.001), but inversely correlated with heartburn (*R* = −0.46, *P* = 0.03). Similarly, the GA-RI was significantly positively correlated with bloating (*R* = 0.60, *P* = 0.002). No other significant correlations were observed between the remaining mapping metrics and individual TSBS indices. Together, the results indicate that heightened gastric slow wave activity after sleeve gastrectomy is associated with bloating, whereas reduced amplitude may predispose to heartburn.

**Table 2:** Pearson’s correlation between patient-reported symptoms and Gastric Alimetry spectral characteristics

Symptom	Metric	*R*	*P*
Bloating	BMI-adjusted amplitude	0.71	< 0.001
Heartburn	BMI-adjusted amplitude	−0.46	0.03
Bloating	GA-RI	0.60	0.002

BMI, body mass index; GA-RI, Gastric Alimetry Rhythm Index.

**Fig. 4:**
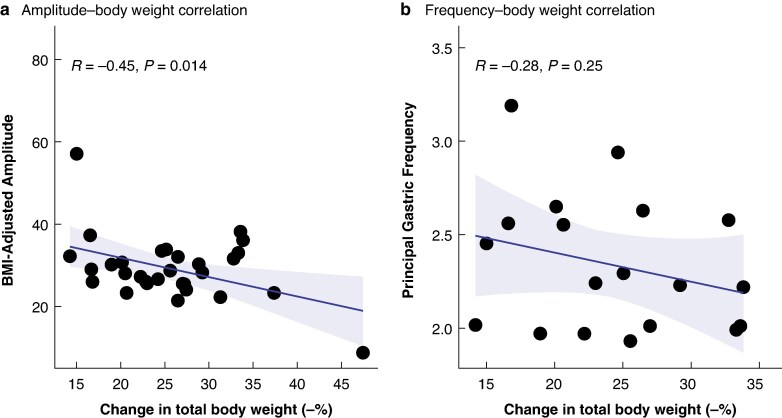
Pearson’s correlation between changes in total body weight and BMI-adjusted amplitude and frequency Correlations between changes in total body weight from referral to follow-up and **a** BMI-adjusted amplitude and **b** principal gastric frequency. Shaded areas indicate 95% confidence intervals. BMI, body mass index.

In addition, lower BMI-adjusted amplitudes at the time of mapping were associated with greater weight reduction from the time of referral (*R* = −0.45; *P* = 0.014), whereas frequency was not correlated with change in weight (*P* = 0.25; *Fig. 4*).

## Discussion

The present body surface gastric mapping study showed consistent changes in gastric electrophysiology occurring after sleeve gastrectomy. Almost all patients were found to have substantially reduced gastric frequency, and a further 59% had one or more additional gastric electrical abnormality in amplitude or rhythm. All metrics were substantially lower than those in the matched controls. Specific relationships were also identified between electrophysiology after sleeve gastrectomy and symptoms of heartburn and bloating, as well as weight loss.

Sleeve gastrectomy is one of the most widely performed bariatric procedures. Although most patients experience excellent outcomes, some develop persistent postoperative symptoms, including reflux, nausea, pain, and excessive food intolerance. Although some chronic symptoms are attributable to mechanical causes (such as stenosis or twisting of the gastric sleeve), a subset of these patients has unexplained symptoms. Previous studies have shown that gastric electrical abnormalities can arise after sleeve gastrectomy^12,13,33^ secondary to resection of the native gastric pacemaker. The present study extends those findings in demonstrating consistent degradation of the intrinsic pacemaker frequency, reduced amplitude, and meal responses associated with gastric symptoms. This investigation was enabled by non-invasive body surface gastric mapping, extending insights from smaller cohorts of patients who had undergone invasive intraoperative mapping^12^.

Early investigations into the surgical effects of disconnecting the normal gastric pacemaker region were conducted using canine models in the early 1970s^34,35^. In one classic study, Kelly and Code^34^ performed a longitudinal bisection of the canine stomach and observed the gastric electrical activity on either side of the bisection using sparse implanted electrodes. Abnormal gastric electrical activity was observed on the lesser curvature half after surgery, at a reduced frequency and with irregular pacemaking^34,35^. Intrinsic frequency gradients have also been identified longitudinally in the stomach from corpus to antrum, and transversely from the greater to lesser curvature, such that loss of the normal dominant highest-frequency pacemaker leads to the breakout of subordinate, lower-frequency, and less-stable pacemaker sites nearer to the lesser curvature^10,36^. These gradients are related to denser Interstitial Cells of Cajal networks occurring along the greater curve, which are resected during sleeve gastrectomy^10^. The results of the present study are consistent with these established physiological data, mostly from classic animal studies, because they similarly reveal the emergence of lower-frequency pacemaking of reduced stability in the human gastric sleeve remnant.

The only previous high-resolution mapping study of sleeve gastrectomy was performed by Berry *et al.*^12^, in which eight patients underwent invasive intraoperative mapping of the stomach before and after sleeve gastrectomy. In that study, abnormal gastric electrical activity was observed, including the presence of retrograde gastric slow wave activity^12^. Berry *et al.*^12^ performed gastric mapping immediately after the resection, and were therefore limited by the invasive nature of the method, meaning that small numbers of patients were studied, and gastric recovery and symptom correlations could not be evaluated. Another notable recent case series was performed by Gharibani *et al.*^33^, who studied 20 patients who had undergone laparoscopic sleeve gastrectomy. In that study, low-resolution electrogastrography was performed, with the authors also finding a reduced frequency of gastric pacemaking activity^33^. Through these and other studies^13^, it is consistently seen that there are significant changes to the gastric conduction system after sleeve gastrectomy. These abnormalities are now expanded through the use of a state-of-the-art gastric mapping technique in the present study, together with symptom correlations. However, further research is still required to confirm whether the associations identified are causal in nature, as well as to address potential confounding factors.

The major advantage of the non-invasive Gastric Alimetry system is that gastric activity can be recorded reliably cutaneously, with major improvements compared with legacy electrogastrography techniques^37^. The system also allows for the simultaneous measurement of symptoms during the test^26^. In the present study, the evaluations were limited to spectral parameters (principal gastric frequency, BMI-adjusted amplitude, and GA-RI); however, in the future, it may also be possible to assess the direction of slow wave propagation non-invasively using body surface gastric mapping^38,39^. This would allow measurement of the directionality of slow wave propagation, which is of interest given previously reported results of retrograde electrical propagation in invasive serosal mapping studies after surgical alterations, including breakout events in the remnant antrum after sleeve gastrectomy^12^, as well as retrograde propagation from the higher intrinsic frequency of the duodenum after a gastroduodenal anastomosis after antrectomy^19^. Gharibans *et al.*^38^ have shown that retrograde propagation can be associated with dyspeptic symptoms and nausea, and disorganized propagation could also be associated with reflux, which is a major complication of sleeve gastrectomy^40^.

Notably, the gastric slow wave amplitude in the present patient cohort was also substantially lower than in the control group, despite adjusting for BMI. This is a novel finding and likely reflects resection of a large mass of Interstitial Cells of Cajal of smooth muscle tissue, such that reduced ionic current flows ultimately arrive at the skin surface. However, a limitation should be noted in that sleeve gastrectomy patients were also only able to eat a smaller-sized meal (half the ‘standard’ meal), which likely also partly accounts for some, but not all, of the group-level amplitude reduction, despite adjusting for BMI^28^. However, previous studies have shown that an approximate 50% meal intake is sufficient to register enough gastric meal response to accurately assess spectral patterns^28^. In addition, lower BMI-adjusted amplitude was associated with greater weight reduction, possibly reflecting the fact that greater gastric resections resulted in a reduced functional sleeve capacity.

The present study found that patients who had undergone a sleeve gastrectomy had significantly worse QoL than the matched cohort across all questionnaires used. Sleeve gastrectomy generally results in an improved QoL *versus* patients’ baselines^2,41,42^. However, there are also studies indicating that a significant proportion of postoperative patients have QoL compromised by persistent symptoms such as reflux^43^. More recent studies have now found a return to baseline QoL results in patients who are followed-up longer term^44^. The present cohort appeared to have a high symptom burden, which could also be due, in part, to selection bias in the cohort, whereby patients with persistent symptoms would be more likely to participate in research.

The findings of the present study indicate that altered gastric slow wave amplitude, rather than frequency, may contribute to the persistent symptoms some patients experience after sleeve gastrectomy. Specifically, higher BMI-adjusted amplitude, associated with a more stable pacemaker rhythm (GA-RI), was associated with bloating, whereas a reduced amplitude was correlated with increased heartburn. These relationships offer a potential electrophysiological explanation for the postoperative symptom burden that extends beyond purely mechanical or anatomical causes, and may point to the emerging role for Gastric Alimetry in clarifying symptom genesis after gastric surgery^22^. Moreover, it is plausible that patients with certain predispositions, such as diabetic gastric dysfunction^44^, are at greater risk of developing disordered motility after sleeve gastrectomy, and may therefore benefit from preoperative functional screening to guide the choice of bariatric procedure. Gastric Alimetry could therefore be incorporated as a tool for patient selection, for example favouring gastric bypass in patients in whom preoperative mapping demonstrates marked dysmotility. Looking ahead, future research may also explore how targeted interventions, such as external gastric pacing or ablation of anomalous pacemaker sites^45,46^, may address pathological gastric rhythms within the sleeve remnant and ultimately improve long-term symptom control.

Despite extensive attempts to recruit a well-matched control cohort, ultimately some minor differences were apparent in age and BMI. To mitigate this, all data were also compared to a normative reference range devised from 110 healthy controls encompassing a range of BMI, age, sex, and ethnicity^23^, and Gastric Alimetry metrics were adjusted for BMI^29^. Furthermore, although consecutive eligible patients were invited to participate from the clinical database, enrolment was ultimately based on patient willingness and availability. This introduces the potential for selection bias, particularly because individuals who were asymptomatic and had returned to full-time work were less likely to participate in research requiring extended time commitment. As such, the study cohort was enriched for patients with significant ongoing symptoms or concerns, which should be considered when interpreting the symptom and QoL findings. Similarly, the cohort exhibited wide variation in both age and time since surgery due to pragmatic recruitment from a real-world patient database. Although this introduced heterogeneity, it also enabled analyses of the persistence of gastric electrophysiological changes over time. Future studies should aim to incorporate more homogeneous subgroups or structured follow-up intervals, ideally with preoperative baseline and longitudinal postoperative gastric mapping, to better delineate temporal patterns and control for confounding variables. The use of complementary motility techniques, such as gastric emptying scintigraphy testing, could also be explored in the future^46^.

Finally, due to the Gastric Alimetry device being used in a variety of different conditions (for example, gastroparesis and other postsurgical gastric motility disorders), the use of validated symptom and QoL questionnaires was standardized to the PAGI-SYM, PAGI-QOL, and EQ-5D-5L. Future studies using validated bariatric-specific questionnaires, including the Bariatric Quality of Life (BQL) Index and Bariatric Analysis and Reporting Outcome System (BAROS), may be more informative^45,47^.

In conclusion, a novel non-invasive gastric mapping device, Gastric Alimetry, was used to assess gastric electrical activity and symptoms after sleeve gastrectomy. The results show that the remodelling and recovery of the gastric pacemaker system are variable, associated with reduced gastric pacemaker frequency, reduced pacemaker stability, and weaker contractile amplitudes that are characteristic after sleeve gastrectomy. Symptom correlations with gastric activity suggest associations that may follow sleeve gastrectomy, and point to the potential role of non-invasive electrophysiology tools in pre- and postsurgical diagnostics, although additional research is required to unlock further insights into this patient population.

## Supplementary Material

zraf140_Supplementary_Data

## Data Availability

The data presented in this study consist of deidentified patient information. The results derived from these data are fully reported within the manuscript. The underlying data are available from the corresponding author upon reasonable request.
