# Supplementary material for: Abnormal gastric electrophysiology following laparoscopic sleeve gastrectomy and associations with symptoms and quality of life
Source: BJS Open. 2025 Dec 1;9(6):zraf140. doi: 10.1093/bjsopen/zraf140 (PMC12667261; doi:10.1093/bjsopen/zraf140)
Supplement: zraf140_Supplementary_Data [file zraf140_supplementary_data.docx]

**TItle**

**Abnormal gastric electrophysiology following laparoscopic sleeve gastrectomy and associations with symptoms and quality of life**

Authors

Tim Hsu-Han Wang^1,2^, Chris Varghese^1^, Sam Robertson^1^, Grant Beban^2^, Nicholas Evennett^2^, Daphne Foong^4^, Vincent Ho^4,5^, Christopher N. Andrews^6^, Stefan Calder^1,3^, Armen Gharibans^1,3,7^, Gabriel Schamberg^1,3^, Greg O'Grady^1,3,7^

**Affiliations**

1. Department of Surgery, University of Auckland, New Zealand
2. Department of Surgery, Auckland City Hospital, Auckland, New Zealand
3. Alimetry Ltd, Auckland, New Zealand
4. School of Medicine, Western Sydney University, Sydney, New South Wales, Australia
5. Department of Gastroenterology and Hepatology, Campbelltown Hospital, Australia
6. Division of Gastroenterology and Hepatology, University of Calgary, Calgary, Alberta, Canada
7. Auckland Bioengineering Institute, The University of Auckland, New Zealand

**Corresponding Author**

Professor Greg O’Grady

Department of Surgery, University of Auckland, New Zealand

Private Bag 92019, Auckland Mail Centre, Auckland 1142, New Zealand

[greg.ogrady@auckland.ac.nz](mailto:greg.ogrady@auckland.ac.nz)

**Supplementary Materials - Index**

| **Supplementary Figures and Tables** |  |
| --- | --- |
| Supplementary Figure 1 | *pag. 2* |

**Supplementary Figure 1**

**
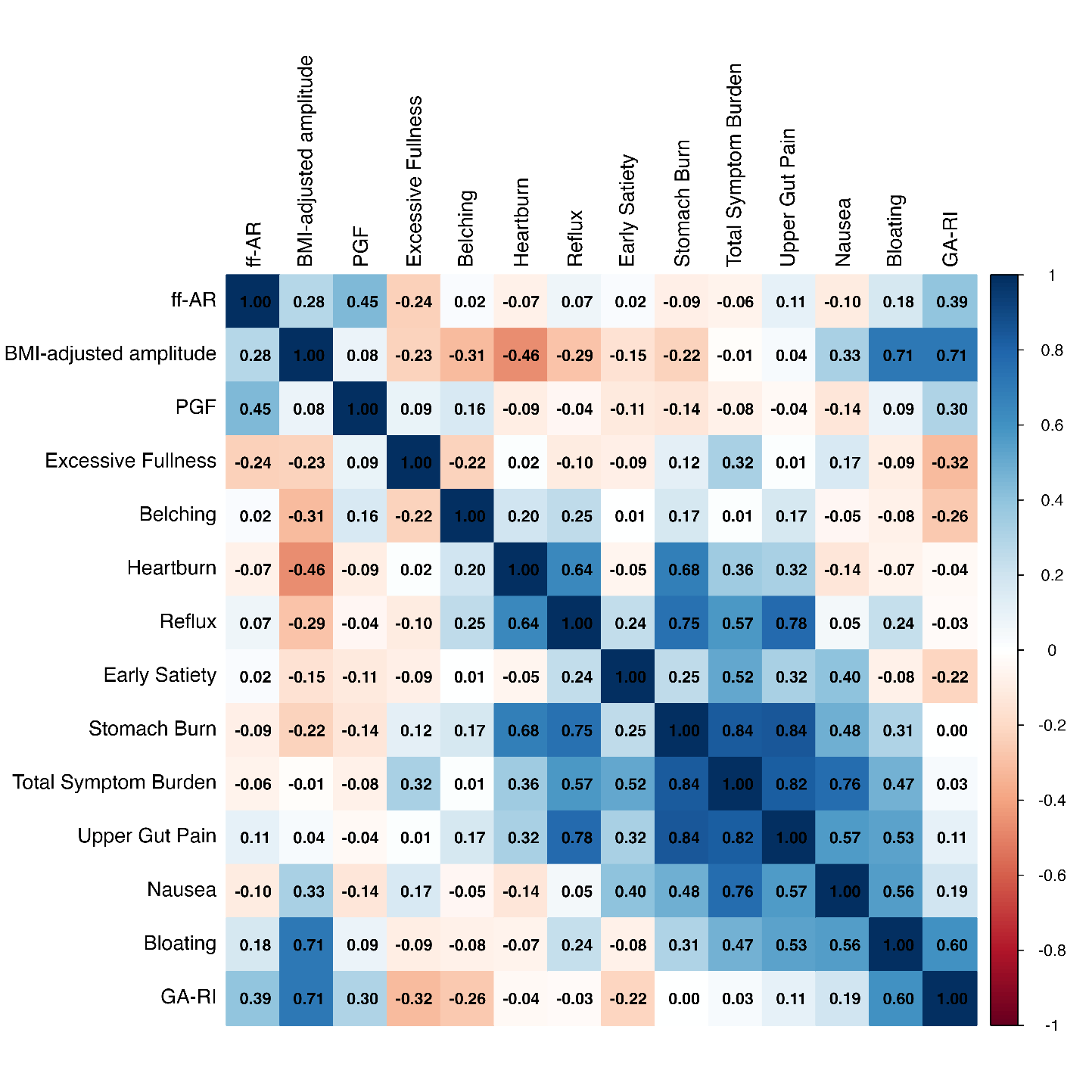
**

**Supplementary Results**

**Supplementary Appendixes**

**Supplementary Figures and Tables**

**References**
